# Insecticide resistance status in *Anopheles gambiae *(*s.l.*) in coastal Kenya

**DOI:** 10.1186/s13071-021-04706-5

**Published:** 2021-04-20

**Authors:** Daniel N. Munywoki, Elizabeth D. Kokwaro, Joseph M. Mwangangi, Ephantus J. Muturi, Charles M. Mbogo

**Affiliations:** 1 Center for Geographic Medicine Research, Coast, Kenya Medical Research Institute, P.O Box 230-80108, Kilifi, Kenya; 2 Department of Zoological Sciences, Kenyatta University, P.O Box 43844-00100, Nairobi, Kenya; 3 Population Health Unit, KEMRI–Wellcome Trust Research Programme, Lenana Road, 197 Lenana Place, P.O Box 43640-00100, Nairobi, Kenya; 4 Crop Bioprotection Research Unit, National Center for Agricultural Utilization Research, Agricultural Research Service, US Department of Agriculture, 1815 N. University, St. Peoria, IL 61604 USA

**Keywords:** *Anopheles*, Insecticide resistance, Kdr, Sodium channel, Coastal Kenya

## Abstract

**Background:**

The rapid and widespread evolution of insecticide resistance has emerged as one of the major challenges facing malaria control programs in sub-Saharan Africa. Understanding the insecticide resistance status of mosquito populations and the underlying mechanisms of insecticide resistance can inform the development of effective and site-specific strategies for resistance prevention and management. The aim of this study was to investigate the insecticide resistance status of *Anopheles gambiae* (*s.l.*) mosquitoes from coastal Kenya.

**Methods:**

*Anopheles gambiae* (*s.l.*) larvae sampled from eight study sites were reared to adulthood in the insectary, and 3- to 5-day-old non-blood-fed females were tested for susceptibility to permethrin, deltamethrin, dichlorodiphenyltrichloroethane (DDT), fenitrothion and bendiocarb using the standard World Health Organization protocol. PCR amplification of rDNA intergenic spacers was used to identify sibling species of the *An. gambiae* complex. The *An. gambiae* (*s.l.*) females were further genotyped for the presence of the L1014S and L1014F knockdown resistance (kdr) mutations by real-time PCR.

**Results:**

*Anopheles arabiensis* was the dominant species, accounting for 95.2% of the total collection, followed by *An. gambiae* (*s.s.*), accounting for 4.8%. *Anopheles gambiae* (*s.l.*) mosquitoes were resistant to deltamethrin, permethrin and fenitrothion but not to bendiocarb and DDT. The L1014S kdr point mutation was detected only in *An. gambiae* (*s.s.*), at a low allelic frequency of 3.33%, and the 1014F kdr mutation was not detected in either *An. gambiae* (*s.s.*) or *An. arabiensis*.

**Conclusion:**

The findings of this study demonstrate phenotypic resistance to pyrethroids and organophosphates and a low level of the L1014S kdr point mutation that may partly be responsible for resistance to pyrethroids. This knowledge may inform the development of insecticide resistance management strategies along the Kenyan Coast.

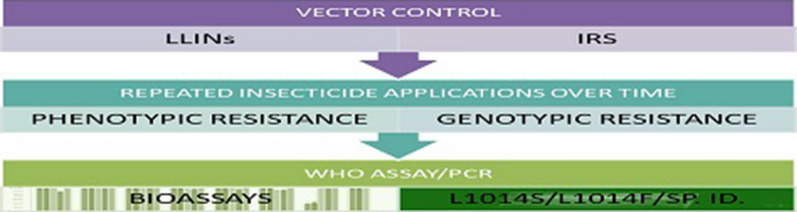

## Background

Malaria is a devastating parasitic disease transmitted by *Anopheles* mosquitoes. Africa bears the heaviest burden of malaria due to presence there of the most virulent malaria parasite, *Plasmodium falciparum*, and the most efficient vectors, *Anopheles gambiae *(*s.s.*) and *An. funestus *(*s.s.*) [1]. Although malaria continues to be a leading cause of mortality in Africa, sustained vector control interventions based on indoor residual spraying (IRS) and long-lasting insecticide-treated nets (LLINs) have contributed to a remarkable decline in malaria-related mortality over the last two decades [2]. Until recently, pyrethroid insecticides were the only class of insecticides approved for use in LLINs, and these pesticides are also commonly used in IRS along with carbamates, organophosphates and organochlorines. Unfortunately, the development of resistance of malaria vectors to these insecticide classes has become a major global threat to their long-term use in the fight against malaria [3–12].

In Kenya, IRS is reserved for small-scale sporadic spraying during epidemics, and this management strategy has only been implemented in the western region of the country [13, 14]. The main insecticide used to supplement deltamethrin (a pyrethroid ester insecticide) in IRS is pirimiphos-methyl (Actellic 300CS; Syngenta Group, Basel, Switzerland), an organophosphate [15]. These insecticides are also used to control agricultural and urban pests, thereby exposing the mosquitoes to persistent selection pressure that eventually results in the selection for insecticide resistance.

Knockdown resistance (kdr) mutations involving the substitution of leucine by serine (L1014S) or phenylalanine (L1014F) at amino acid position 1014 is the main mechanism of resistance to pyrethroids in malaria vectors [16], although metabolic enzymes have also been known to play a role in resistance to pyrethroids [17, 18]. Earlier studies reported that the L1014S mutation was restricted to East Africa while the L1014F mutation was widely distributed in West and Central Africa [19]. However, recent studies have shown that both the L1014S and L1014F kdr mutations co-exist in East and West Africa [20, 21]. Conversely, resistance to carbamates and organophosphates results from overexpression of non-specific esterase enzymes and/or alteration of acetylcholinesterase (AChE) due to a single glycine to serine amino acid substitution at position 119 of the AChE gene [22, 23].

Defining the prevalence of insecticide resistance in malaria vectors and the underlying mechanism(s) of resistance in different ecological settings is necessary for the development of rational strategies for insecticide use and resistance management. The objective of this study was to investigate the resistance status of *An*. *gambiae *(*s.l.*) and elucidate the kdr mutation in coastal Kenya. Historically, *An. gambiae *(*s.s.*) and *An. funestus* were the main vectors of malaria in coastal Kenya, but the widespread use of LLINs has led to a major shift in malaria vectors in favor of *An. arabiensis*, an exophilic mosquito species that has had less contact with LLINs [24–28]. Recent reports in Kilifi county [25] and the neighboring Kwale county [26, 27] have revealed a low frequency of both the kdr allele and pyrethroid phenotypic resistance in this region. In addition, malaria is endemic in Kilifi county, with 91% of households having reported possessing at least one LLIN and 51% having universal ITN coverage achieving the proposed target of ≤ 2 people per LLIN [29]. The findings of this study will provide critical knowledge that may contribute to improved strategies for malaria control in Africa.

## Methods

### Study area

This study was conducted in Kilifi county, located along the north coast of Kenya. The county is situated north and northeast of Mombasa county, which also has a coastline on the Indian Ocean, with Taita Taveta county to the west, Tana River county to the east and Kwale county to the southwest. Kilifi county covers an approximate area of 12,246 km^2^, and the population at risk of malaria and other mosquito-borne diseases is approximately 1.5 million [30]. The study sites have been described in detail elsewhere [24, 31]. *Anopheles gambiae* (*s.l.*) and *An. funestus* (*s.l.*) are the key malaria vectors in the area. They occur throughout the year, with peak occurrence during the rainy season [24]. Coastal Kenya usually experiences two rainy seasons each year: short rains falling from October to November and long rains from April to July. The mean annual rainfall ranges between 300 mm in the hinterland and 1300 mm in the coastal belt. The mean daily temperature varies from 21 °C to 30 °C [32].

**Fig. 1:**
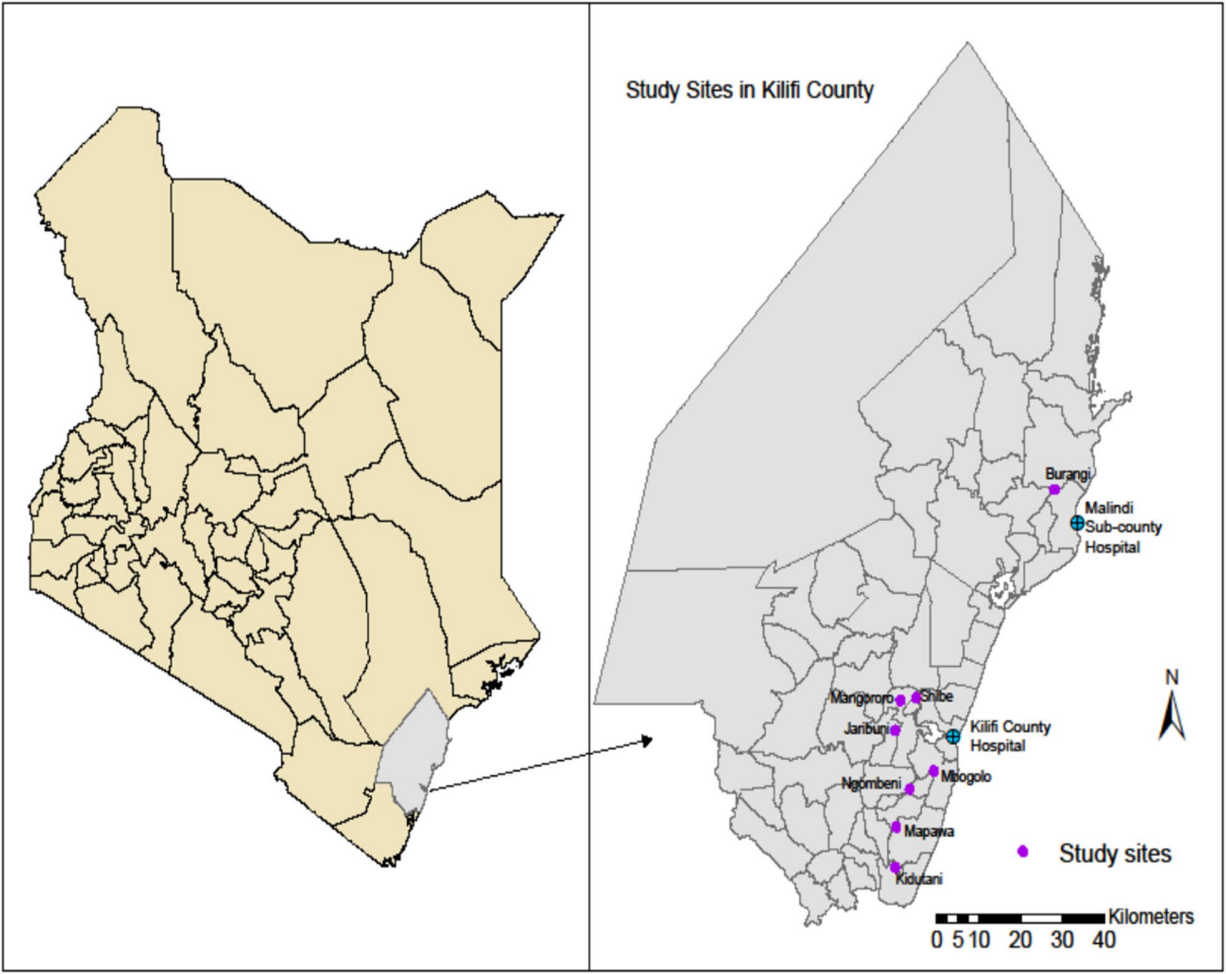
Map of Kenya and Kilifi county showing the study sites location

Larval mosquito sampling was carried out in eight randomly selected study sites (Fig. 1), of which seven were in Kilifi subcounty, namely Ngombeni (3.73208°S, 39.76491°E), Mbogolo (3.69806°S, 39.81706°E), Jaribuni (3.62054°S, 39.73354°E), Kidutani (3.88209°S, 39.71819°E), Shibe (3.55840°S, 39.77921°E), Mapawa (3.80452°S, 39.73641°E) and Mangororo (3.56366°S, 39.74507°E), and one, Burangi (3.09828°S, 40.04817°E), was in Malindi subcounty.

Selection of the study sites was based on high coverage with LLINs, and abundance and ease of accessibility to mosquito breeding sites.

### Larval mosquito collection, transportation and rearing

Larval mosquito collections were done in June (during long rains), August (dry season) and November (short rains) 2013 and in July 2014 (immediately after the long rains). The collections were spread across the various seasons to maximize the collection of the different malaria vectors in the study sites. Larvae were sampled from stagnant water bodies selected in each study site using the standard dipping technique [33]. In each study site, six larval habitats were sampled once per week. The habitats included sand pits, ponds, roadside ditches, marshes, shallow wells and river banks. Anopheline larvae were transferred into Whirl-pak® bags (Thermo Fisher Scientific, Waltham, MA, USA) and transported to the insectary at Kenya Medical Research Institute (KEMRI), Center for Geographic Medicine Research, Coast Kilifi.

Tetramin® (Tetra Werke, Melle, Germany) baby fish food was used as the larval rearing diet. The insectary was maintained at a constant temperature of 25–27 °C and relative humidity of 74–82%. The larval pans were monitored daily, and the collected pupae transferred into plastic cups in emergence cages. Newly emerged adults (F0) were identified using morphological characteristics [34]. The adults were then kept as same-age cohorts for insecticide susceptibility testing.

### World Health Organization susceptibility bioassay tests

Non-blood-fed female *An. gambiae * (*s.l.*) aged between 3 and 5 days and reared from field-collected larvae were used in the susceptibility bioassays. Each insecticide susceptibility test was performed using 25 mosquitoes in four replicates and two controls. The insecticides used and their concentrations included deltamethrin (0.05%), permethrin (0.75%), dichlorodiphenyltrichloroethane (DDT, 4%), fenitrothion (1%) and bendiocarb (0.1%), prepared at Universiti Sains Malaysia [35]. The negative control included 25 mosquitoes collected from each study site and exposed to untreated filter papers. In addition, laboratory-raised, susceptible *An. gambiae *(*s.s*) Kisumu strain were exposed to insecticide-treated filter papers as a measure of quality assurance. The knockdown time (KDT) of each insecticide was recorded every 10 min for 1 h. Mosquitoes were then moved to a recovery tube and provided with 10% sucrose. Final mortality was recorded after 24 h.

After recording mortality at 24 h post-exposure, those mosquitoes still alive were killed by freezing and together with a 20% randomly selected subset of the dead samples per study site stored individually in labeled Eppendorf tubes (Eppendorf Co., Hamburg, Germany). The specimens were then preserved at − 80 °C for later molecular analysis.

### DNA extraction and species identification

Genomic DNA was extracted from the whole body of the *An. gambiae *(*s.l.*) using an ethanol precipitation method [36]. Conventional PCR amplification of ribosomal DNA intergenic spacers was used to differentiate the sibling species of the *An. gambiae* complex [37].

### Detection of kdr genotype mutations

Knockdown resistance was tested using 192 mosquitoes (59 alive and 133 dead) after performing the pyrethroid insecticide susceptibility tests; 20 *An. gambiae *(*s.s.*) Kisumu strain were used as controls. Both the L1014S (leucine to serine substitution) kdr allele originally detected in East Africa [16] and the L1014F (leucine to phenylalanine substitution) kdr allele from West Africa [19] were tested. Real-time PCR was used to determine the genotype at amino acid position 1014 of the voltage-gated sodium channel, following the methods of Bass et al. [38] as modified by Mathias et al. [39]. Each PCR reaction was conducted in a 10-µl volume containing 5.0 µl 2× TaqMan mix (TaqMan® Gene expression Master Mix [Thermo Fisher Scientific), 0.5 µl of forward primer, 0.5 µl reverse primer [for either kdr-east or kdr-west], 0.4 µl of each probe, 1.0 µl of template DNA and 2.6 µl of PCR grade water [ddH_2_O]). Samples were genotyped for the wild-type (susceptible) allele using the probe 5′-CTTACGACTAAATTTC-3′ and for the L1014S kdr allele using the probe 5′-ACGACTGAATTTC-3′. The primer sequences used for this study were 5′-CATTTTTCTTGGCCACTGTAGTGA-3′ (forward) and 5′-CGATCTTGGCCATGTTAATTTGCA-3′ (reverse). Controls loaded in the last four wells of the 96-well PCR plate included FAM (fluorescent label)-positive control, HEX (fluorescent label)-negative control, non-template control (NTC) and buffer. The PCR was carried out in a Strategene® MxPro 3000 Real-Time PCR system (Strategene, Agilent Technologies, Inc., La Jolla, CA, USA) at the following temperature profile: an initial denaturation step at 95 °C, 10 min; followed by 92 °C (for kdr-east) or 95 °C (for kdr-west)/15 s, 60 °C/1 min, for 40 cycles; with a final extension step at 72 °C for 10 s.

### Data analysis

Resistance was obtained using the World Health Organization criteria, with mortality rates of 98–100% indicating susceptibility, 90–97% suggesting possible resistance that needs further investigation and ≤ 90% indicating resistance, respectively [35]. KDT_50_ and KDT_95_ (time [in minutes] to knock down 50 and 95% of mosquitoes, respectively) were estimated using probit analysis [40]. A post-hoc comparison test with the Bonferroni correction test was performed to compare the proportions of each mosquito species among the study sites. The scatter-plots were retrieved to assess the kdr mutation and the frequency of the resistance allele determined from endpoint fluorescence using the MxPro software (Agilent Technologies, Santa Clara, CA, USA). Final data were captured in Microsoft Excel 2010 (Microsoft Corp., Redmond, WA, USA) and analyzed using the R statistical package, version 3.3.2 (R Foundation for Statistical Computing, Vienna, Austria).

## Results

### Sample size and species composition

A total of 3683 *An. gambiae * (*s.l.*) adult females were collected as larvae and used for the susceptibility bioassays from the eight villages: Jaribuni (500), Shibe (500), Kidutani (475), Ng’ombeni (490), Mapawa (475), Mbogolo (455), Burangi (500) and Mangororo (288). PCR analyses showed that *An. arabiensis* (91.49%) and *An. gambiae *(*s.s*) (5.02%) were the only sibling species of *An. gambiae * (*s.l.*). No amplification occurred in the remaining 3.49% of samples (Table 1). Mosquitoes from Mangororo, Shibe and Mbogolo were predominantly *An. arabiensis* (Mangororo: 100%; Shibe: 96.8%; Mbogolo: 99.01%). A post hoc comparison test with Bonferroni correction revealed that the proportions of *An. gambiae *(*s.s*) and *An. arabiensis* species among the study sites were significant (*Z* = − 3.31, *P* = 0.001). No *An. funestus* were collected in this study.

**Table 1:** Proportions of *Anopheles gambiae *(*s.s*) and *An. arabiensis* in the eight study sites, Kilifi county

Study site	*n*	*An. gambiae *(*s.s*)	*An. arabiensis*	Not amplified
Jaribuni	172	12 (6.98%)	148 (86.04%)	12 (6.98%)
Kidutani	124	6 (4.84%)	114 (91.94%)	4(4.00%)
Mbogolo	101	0 (0.00%)	100 (99.01%)	1 (0.99%)
Ngombeni	123	18 (14.63%)	102 (82.93%)	3 (2.44%)
Shibe	124	0 (0.00%)	120 (96.77%)	4 (3.23%)
Mapawa	108	5 (4.63%)	95 (87.96%)	8 (7.41%)
Burangi	163	8 (4.91%)	153 (93.86%)	2 (1.23%)
Mangororo	60	0 (0.00%)	60 (100%)	0 (0.00%)
Total	975	49 (5.02%)	892 (91.49%)	34 (3.49%)

*n*, Number of *An. gambiae *(*s.l.*) processed for species identification

### Insecticide susceptibility bioassays

*Anopheles gambiae *(*s.s*) Kisumu strain were susceptible to all of the insecticides tested (deltamethrin, permethrin, DDT, bendiocarb and fenitrothion) with 100% mortality. The negative control did not show any mortality; therefore, Abbott’s formula was inapplicable to correct for the natural causes of mortality in this study.

**Fig. 2:**
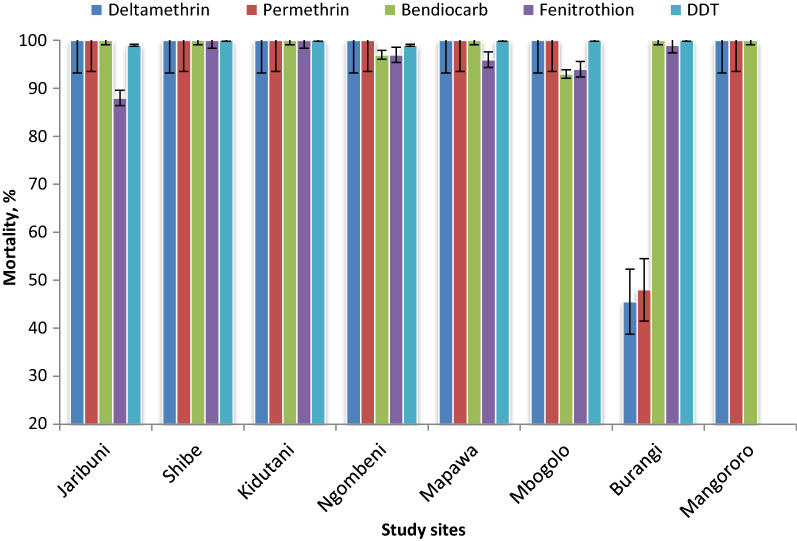
Results of bioassay tests of female *Anopheles *from eight study sites exposed to various insecticides.* DDT* Dichlorodiphenyltrichloroethane

Figure 2 shows the bioassay susceptibility status of *An. gambiae * (*s.l.*) to the different insecticides used in this study. Deltamethrin resistance in *An. gambiae * (*s.l.*) was only recorded in one of the eight study sites (Burangi: *n* = 100, mortality 45.5%). Coincidentally, permethrin resistance was also reported in the same study site (*n* = 100, mortality 48%). However, full susceptibility to both permethrin and deltamethrin in *An. gambiae * (*s.l.*) was indicated in all of the other study sites (mortality 100%). The *An. gambiae *(*s.l.*) populations tested indicated full susceptibility to DDT (*n* = 750, mortality range 99–100%) in the seven study sites tested.

Adult mosquitoes were resistant to fenitrothion in Jaribuni (*n* = 100, mortality 88%) and showed possible resistance at Ngombeni, Mapawa and Mbogolo with a test mortality range of 94–97%. However, mosquitoes from Burangi, Kidutani and Shibe indicated full susceptibility to fenitrothion. The mosquitoes tested were highly susceptible to bendiocarb in six of the eight study sites, i.e. Jaribuni, Shibe, Kidutani, Mangororo, Burangi and Mapawa, with test mortality of 100%. However, the possibility of resistance that requires further investigation was recorded against bendiocarb in Mbogolo (*n* = 75, mortality 93%) and Ng’ombeni (*n* = 90, mortality 97%).

### Knockdown time (KDT_50_) at 95% CI and KDT_50_ ratio

Most of the knockdown resistance ratios (KDT_50_ ratio: KDT_50_ of the test population to that of the control [Kisumu strain]) were within the *An. gambiae *(*s.s.*) Kisumu strain susceptible range of 1.0 to 1.7 and varied among study sites, as indicated in Table 2. The KDT_50_ ratio for deltamethrin could not be determined in Burangi due to the high resistance levels. However, in the other study sites, the KDT_50_ ratio was 1.1–1.9 fold compared to Kisumu strain. The mosquito population from Burangi also recorded resistance to permethrin with a KDT_50_ ratio of 3.3. The resistance ratio indicated potential resistance to permethrin in Kidutani (KDT_50_ ratio: 2.0) which requires further investigation. The KDT_50_ ratio was 1.0- to 1.7-fold in the remaining six study sites: Jaribuni, Shibe, Ngombeni, Mapawa, Mbogolo and Mangororo.

**Table 2:** Knockdown resistance ratio of mosquito populations (*An. gambiae * [*s.l.*]) to different insecticides

Insecticide	Population	KDT_50_ (in min)	95% CI	KDT_50_ ratio
Deltamethrin	Jaribuni	18	40.9–55.2	1.1
	Shibe	16	43.9–58.1	0.9
	Kidutani	18	42.9–57.3	1.1
	Ngombeni	23	41.9–56.2	1.4
	Mapawa	32	40.8–61.1	1.9
	Mbogolo	25	39.8–60.2	1.5
	Burangi	–^a^	–^a^	–^a^
	Mangororo	30	39.8–60.2	1.8
Permethrin	Jaribuni	18	39.9–60.2	1.0
	Shibe	21.5	37.9–58.2	1.2
	Kidutani	35.5	38.9–59.2	2.0
	Ngombeni	30	40.8–61.1	1.7
	Mapawa	20	39.8–60.2	1.1
	Mbogolo	27	38.9–59.2	1.5
	Burangi	60	37.9–58.2	3.3
	Mangororo	30	39.8–60.2	1.7
Dichlorodiphenyltrichloroethane (DDT)	Jaribuni	30.5	40.9–55.2	2.1
	Shibe	15	37.9–58.2	1.0
	Kidutani	18	39.8–60.2	1.2
	Ngombeni	27.3	39.8–60.2	1.9
	Mapawa	31.5	39.8–60.2	2.2
	Mbogolo	25	39.8–60.2	1.7
	Burangi	24.4	36.9–57.2	1.7
Bendiocarb	Jaribuni	19.2	43.9–58.1	1.0
	Shibe	19.3	39.8–60.2	1.0
	Kidutani	22	39.8–60.2	1.2
	Ngombeni	26.9	39.8–60.2	1.4
	Mapawa	30	39.8–60.2	1.6
	Mbogolo	23	37.9–58.2	1.2
	Burangi	24	37.9–58.2	1.3
	Mangororo	30	40.8–61.1	1.6
Fenitrothion	Jaribuni	46	37.9–58.1	2.1
	Shibe	23.5	39.8–609.2	1.1
	Kidutani	40	42.8–63.1	1.9
	Ngombeni	31	38.9–59.2	1.5
	Mapawa	21.5	37.9–58.2	1.0
	Mbogolo	46.3	37.9–58.2	2.2
	Burangi	36	36.9–57.2	1.7

CI, Confidence interval; KDT_50_, time taken for 50% of the test population to be knocked down; KDT_50_ ratio, KDT_50_ of the test population to that of the control (Kisumu strain)

^a^Lower limit estimation not possible due to a large *g* value [40]

The KDT_50_ ratio for DDT, fenitrothion and bendiocarb compared to that for the Kisumu strain ranged from 1.1- to 2.2-fold in all the study sites. The mosquitoes were susceptible to bendiocarb in all the study sites (KDT_50_ ratio 1.0–1.6). Possible resistance was recorded in Jaribuni for both DDT (KDT_50_ ratio 2.2) and fenitrothion (KDT_50_ ratio 2.1). The KDT_50_ ratio for both DDT and fenitrothion was 2.2 in both Mbogolo and Mapawa, indicating possible resistance.

### Knockdown resistance status

A total of 192 randomly selected samples comprising of *An. arabiensis*,* An. gambiae* (*s.s.*) and Kisumu strain were genotyped for the kdr mutation. Neither L1014F nor L1014S kdr allelic genes were observed in *An. arabiensis* species. The kdr allelic frequency in *An. gambiae*, *An. arabiensis* and* An. gambiae* (*s.s.*) populations is shown in Table 3. Upon exposure to pyrethroids for 24 h, a total of 15 *An. gambiae*, *An. arabiensis* and* An. gambiae* (*s.s.*) were identified with the resistant phenotype, while 34 had the susceptible phenotype. L1014S allelic genes were identified in only four *Anopheles gambiae*, *An. arabiensis* and* An. gambiae* (*s.s.*), of which two were homozygous (RR) and two were heterozygous (RS) for the trait. Only one of the 15 phenotypically resistant mosquitoes was observed to be genotypically resistant; this mosquito was heterozygous for L1014S characteristic. The remaining An. gambiae, *An. arabiensis* and* An. gambiae* (*s.s.*). mosquitoes genotyped were recessive (SS).

**Table 3:** Knockdown resistance allelic frequency in *An. gambiae *(*s.s.*) populations from Kilifi county, coastal Kenya

Mosquito status^a^	*N*	L1014S allele^b^			*F*^c^
		RR	RS	SS	
Resistant to pyrethroids	15	0	1	14	0.0333
Susceptible to pyrethroids	34	2	1	33	0.0735

*N*, Number of mosquitoes genotyped

^a^Resistant refers to mosquitoes alive after 24 h of exposure to pyrethroid pesticide; susceptible indicates dead mosquitoes after 24 h of exposure to pyrethroid pesticide

^b^L1014S allele: leucine to serine substitution knockdown resistance (kdr) allele. RR, Homozygous resistant allele; RS, heterozygous resistant allele; SS, susceptible allele

^c^ *F*, kdr allele frequency

## Discussion

This study has shown that *Anopheles arabiensis* is the most dominant species in the eight study sites sampled in Kilifi county. An earlier study reported that *Anopheles gambiae *(*s.s.*) was the dominant subspecies along the Kenyan coast and other regions of the country [24]. However, a shift in malaria vector composition has recently been documented that coincides with the scaling up of vector control interventions, especially the ongoing widespread use of insecticide-treated nets (ITNs) along the Kenyan coast [31]. The increase in populations of *An. arabiensis* and declining populations of *An. gambiae *(*s.s.*) may be a result of their contrasting ecological behavior. *Anopheles gambiae *(*s.s.*) has anthropophilic, endophagic and endophilic tendencies [41, 42]. This means relatively more hours spent indoors and, consequently, longer contact hours with LLINs, which may have contributed to its population decrease. In contrast, *An. arabiensis* is both exophilic and zoophilic [43–45], and this limits its contact with LLINs and the insecticides used for IRS [46]. The observed shift in species composition in favor of *An. arabiensis* calls for an integrated vector management (IVM) approach targeting both indoor and outdoor control of malaria vectors.

Mosquito populations from one of the eight study sites (Burangi) were resistant to both deltamethrin and permethrin and had high median KDT compared with the other study sites. These findings suggest that resistant genes are localized and that vector control management strategies should be established to prevent their spread and preserve the efficacy of the current vector control tools. Earlier studies along the Kenyan coast and neighboring Tanzania reported the presence of pyrethroid resistance characterized by a high median KDT and mortality levels ranging from 62.38 to 93% following pyrethroid exposure [25, 28, 47]. In some West African countries, such as Ivory Coast and Burkina Faso, pyrethroid resistance has been found to be much more intense, with high KDT_50_ ratios and mortality rates of ≤ 40% reported following exposure to pyrethroids [48–52]. High KDT_50_ values in the test populations indicate the presence of the kdr mechanism of resistance [53]. The high resistance levels might be attributed to selection for insecticide resistance due to increased use of LLINs in vector control programs [35, 54, 55]. Based on KDT_50_ values, we noted potential resistance to DDT in Jaribuni and Mapawa villages that might partly be linked to cross-resistance from pyrethroids [56] or the presence of recessive genes in the mosquito population [25]. These two insecticide classes (pyrethroids and organochlorines) have the same mode of action and thus pose a threat of cross-resistance.

Mosquito samples from Jaribuni were resistant to fenitrothion based on both mortality rate and the KDT_50_ ratio. In addition, potential resistance to both fenitrothion and bendiocarb was recorded in Mbogolo and Ngombeni. However, susceptibility to both fenitrothion and bendiocarb was detected in all other study sites. This resistance may be due to selection pressure resulting from the contamination of the larval habitats with carbamates and organophosphates used in agriculture [57] as well as exposure of adult mosquitoes to insecticides during either sugar-feeding or outdoor resting. Agricultural systems provide ideal habitats for mosquito breeding [27]. Earlier studies conducted in Kenya reported the use of fenitrothion and bendiocarb in agricultural settings and linked them to resistance [58, 59]. Fenitrothion (organophosphate) and bendiocarb (carbamate) have been proposed for IRS use to control pyrethroid-resistant mosquitoes [60]. Therefore, the evolution of resistance to these insecticides threatens their use in IRS and as substitute to pyrethroids in public health systems.

The occurrence of phenotypic resistance to pyrethroids (deltamethrin and permethrin) may indicate the presence of target site insensitivity. Our findings that no kdr alleles were detected in *An. arabiensis* are consistent with results from previous studies in western and coastal Kenya [28, 39], suggesting other underlying resistance mechanisms as the cause of phenotypic resistance. However, the L1014S kdr allele was detected in *An. gambiae *(*s.s.*) with an allelic frequency of 3.33% in the resistant test population. The low allelic frequency of the L1014S kdr gene is consistent with results reported in other studies in the neighboring Kwale county [26, 28]. In contrast, other studies have indicated a high frequency and wide distribution of the L1014S mutation in *An. arabiensis* in western Kenya [61, 62]. These variations in the frequency of the resistance allele may be either due to movement of the mutant genes from their original selection pressure region or DNA deletion in a given genome in a mosquito population [39, 63, 64]. Although target site resistance had been detected earlier in the neighboring Kwale county along the coastal Kenya, in practice this may not mean that the mutant genes originated from there. However, it is evident that the percentage allelic frequency is clearly increasing. Therefore, further studies are needed to ascertain the origin of the mutant genes and facilitate development of strategies to mitigate their rapid spread.

The low kdr allele frequency compared to the high phenotypic resistance observed in pyrethroids is indicative of other underlying resistance mechanisms. Indeed, metabolic-based resistance most likely plays a bigger role in insecticide resistance than target site resistance [65]. Several studies have documented the contribution of metabolic resistance enzymes in malaria vectors to pyrethroids, organophosphates and carbamates [4, 26, 62, 63, 66–68]. Knockdown resistance is considered to be a weak form of resistance compared to metabolic-based resistance. Therefore, vector control failure is likely when kdr occurs along with metabolic resistance [69]. Nevertheless, the increasing kdr allele frequency coupled with metabolic-based resistance reported in the neighboring Kwale county [28] focuses attention on the risk of subsequent failure of current vector control interventions in coastal Kenya. Therefore, further investigation on the evolution of insecticide resistance in *An. gambiae * (*s.l.*) in Kilifi county is vital.

## Conclusions

This study reported phenotypic resistance to selected pyrethroids and organophosphate insecticides in malaria vectors along the Kenyan coast. The occurrence of the L1014S allele in *An. gambiae *(*s.s.*) at a low frequency was also documented. These findings highlight the need for regular entomological surveillance and monitoring of insecticide resistance and for investment in new vector control strategies that can supplement or even replace the use of synthetic insecticides. This effort may inform development and implementation of an IVM approach targeting all mosquito life stages with diverse vector control interventions to limit the spread of insecticide resistance and preserve the efficacy of existing insecticides.

## Data Availability

All data generated and supporting the conclusions of this manuscript has been included in the article. Raw data and materials are available from the corresponding author upon request.

## Abbreviations

AchE: Acetylcholinesterase
ddH_2_O: PCR grade water
DDT: Dichlorodiphenyltrichloroethane
IRS: Indoor residual spraying
ITN: Insecticide-treated net
IVM: Integrated vector management
KDT: Knockdown time
kdr: Knockdown resistance
LLINs: Long-lasting insecticide-treated nets
NTC: Non-template control
